# Outcomes of severe traumatic brain injury at time of discharge from tertiary academic hospitals in Bloemfontein

**DOI:** 10.7196/AJTCCM.2020.v26i2.057

**Published:** 2020-06-15

**Authors:** S D Maasdorp, C Swanepoel, L Gunter

**Affiliations:** 1 Division of Pulmonology and Critical Care, Department of Internal Medicine, Faculty of Health Sciences, University of the Free State, Bloemfontein, South Africa; 2 Trauma Department, Pelonomi Tertiary Hospital, Bloemfontein, South Africa; 3 Intensive Care Unit, Pelonomi Tertiary Hospital, Bloemfontein, South Africa

**Keywords:** Traumatic brain injury, TBI, outcomes, rehabilitation

## Abstract

**Background:**

Despite the condition being a major public health concern, limited data are available regarding survival rates and the requirement for post-hospitalisation support of patients with traumatic brain injury (TBI) in South Africa (SA).

**Objectives:**

To describe the clinical profile and in-hospital outcomes of patients with TBI at intensive care units (ICUs) of tertiary referral hospitals in the Free State Province, SA, between 2013 and 2017.

**Methods:**

This retrospective descriptive study of patients with TBI was conducted at Pelonomi Tertiary and Universitas Academic Hospitals. Patients’ demographic information and variables such as mechanism and type of injury, Glasgow Coma Scale (GCS) prior to ICU admission, neurosurgical intervention, duration of stay in ICU and hospital, GCS and final outcome at discharge were recorded.

**Results:**

The 138 patients included in the final data analysis had a median (range) age of 30.5 (13 - 70) years, with a male predominance of 82.6%. The median lengths of stay in ICU and hospital were 6 and 16 days, respectively. Outcomes data showed that 65.9% of patients survived until discharge from hospital. Of patients whose GCS could be determined at discharge, 52.8% were deceased, 7.9% were in a persistently vegetative state, 11.2% had severe and 13.5% moderate disability, and 14.6% had a good recovery.

**Conclusion:**

TBI is associated with high mortality and morbidity. The lack of post-hospitalisation rehabilitation, and support for patients and their caregivers, requires urgent redress.

## Background

Traumatic brain injury (TBI) can be defined as 'an alteration in brain function manifest with confusion, altered level of consciousness, seizure, coma, or focal sensory or motor neurologic deficit resulting from blunt or penetrating force to the head.'^[1]^ It poses a major public health problem, with an estimated annual hospitalisation rate of 90.5 per 100 000 population in the USA,^[2]^ and more than 200 per 100 000 in Europe.^[3]^ Although there is a paucity of epidemiological studies regarding the incidence of TBI in South Africa (SA), an incidence of 316 per 100 000 per year has been reported.^[4]^

TBI is generally graded as mild if the Glasgow Coma Scale (GCS) score is 13 - 15, moderate if 9 - 12 and severe if 3 - 8 after resuscitation.^[5]^ Since the brain is enclosed in a non-deformable skull, any increase in intracerebral volume as a result of haemorrhage or oedema can cause a significant increase in intracranial pressure. Raised intracranial pressure can potentially lead to reduced cerebral perfusion pressure (CPP) associated with cerebral hypoxia and ischaemia, ultimately resulting in permanent neurological impairment. The management of patients with severe TBI therefore mainly entails reducing intracranial pressure by draining any haematomas that exert a pressure effect on the underlying brain parenchyma, but it also aims to prevent secondary brain injury by means of optimising CPP and via oxygenation of brain tissue.^[6]^ This can be achieved by intubating and ventilating the patient, providing isotonic fluid and vasopressor therapy to ensure normotension, maintaining normothermia and preventing either hypo- or hyperglycaemia.

In SA, most people suffering TBIs are young and otherwise healthy adults who may survive the initial injury, but are often left with severe cognitive or functional impairment.^[7]^ Furthermore, the reality in resource-constrained countries such as SA is that post-hospital discharge rehabilitation or long-term care facilities for patients with severe neurological disabilities are not readily available in the public healthcare sector.^[8]^

Andrew *et al*. ^[9]^ report alarming information on the admission of patients with TBI to a public rehabilitation centre in the Western Cape Province. According to previously unpublished statistics, only 2.4% (n=16) of 654 patients with TBI treated at Groote Schuur Hospital in 2009 were admitted to a rehabilitation facility. In 2013, a total of 2 851 patients with TBI were treated at Groote Schuur and Tygerberg Hospitals, of whom 2.9% (n=82) were discharged from hospital to a rehabilitation centre.^[9]^ Webster *et al*.^[8]^ reported that <9% of all patients with TBI were admitted to the Western Cape Rehabilitation Centre for the 5-year period 2008 - 2012. No similar information is currently available for the Free State Province.

It has been argued that SA patients are not hospitalised for a sufficient period of time after sustaining a TBI. Because of the shortage of specialised rehabilitation facilities, which negatively influences outcome, patients have to be cared for by their families, who are ill equipped for the task and lack critical knowledge and support.^[9]^

It is difficult to predict the eventual residual neurological impairment or disability of a patient with severe TBI at the time of presentation to hospital, since improvement in neurological function can occur up to a year after the initial injury, depending on its severity.^[10]^ From an ethical point of view, one can argue that post-hospitalisation rehabilitation of patients with impaired cognitive or functional abilities, especially in resource-constrained environments, is especially important in view of the psychosocial and economic impact on society if the outcome is one of functionally dependent or economically unproductive individuals.

The aim of this study was therefore to describe the clinical profile and outcomes of patients with TBI who were admitted to tertiary hospital intensive care units (ICUs) in the Free State Province of SA between 2013 and 2017, with a view to determine the need for functional rehabilitation of such patients at the time of discharge from hospital.

## Methods

The study was conducted as a retrospective clinical audit of patient hospital files. The primary objectives were to describe the clinical profile and in-hospital outcome of patients with TBI who were admitted to the Pelonomi Tertiary and Universitas Academic Hospitals in Bloemfontein during the 5-year period 2013 - 2017. This specific period was selected because we expected that hospital records would have been stored at the hospital archives for at least 5 years, and that it would pose a challenge to obtain complete hospital files prior to 2013. All adult patients ≥18 years of age who were admitted to the ICUs of either Pelonomi Tertiary or Universitas Academic Hospitals between 2013 and 2017 were included in the study. Patients were excluded if the hospital files were incomplete or adequate clinical data were not available.

Data forms were designed using a Microsoft Excel spreadsheet to capture the following variables: demographics; mechanism of injury; type of head injury; concomitant injuries; GCS on ICU admission; neurosurgical interventions performed; outcomes data, including length of stay in ICU and in hospital until discharge or demise; GCS at time of discharge from ICU and hospital (where recorded in patient files); and Glasgow Outcome Scale (GOS) score at the time of discharge from hospital in those who survived (if this was recorded, or could be inferred from patient files). The GOS is a standardised 5-point scale that is widely used to determine and document the neurological outcome of patients after sustaining a TBI.^[11]^ As per the GOS, patient outcomes at the time of discharge from hospital after the initial trauma were classified as follows: deceased; persistent vegetative state (≤8/15); severely disabled (9 - 12/15); moderately disabled (13 - 14/15); or good recovery (15/15). For the purpose of this study, the GOS was determined by reviewing clinical records of patients at the time of discharge from hospital for classification of outcome.

Data collection and compiling the study report were conducted between 1 January 2019 and 31 October 2019. All patient information was kept anonymous and confidential. A pilot study with three patients was performed to test the data collection sheet, and since no changes to this were required, the results of this pilot study were included in the main study for statistical analysis. Descriptive statistics, namely medians and percentiles, were calculated for continuous data. Frequencies and percentages were calculated for categorical data.

Approval to conduct the study was obtained from the Ethics Committee of the Faculty of Health Sciences, University of the Free State (ref. no. UFS-HSD2018/0804/2711) and the Free State Province Department of Health.

## Results

The hospital files of 138 patients were included in the study. Findings on demographic and clinical variables are summarised in Table 1. The median age was 30.5 years, and patients were predominantly male (n=114; 82.6%). Assaults (n=57; 41.3%) and motor vehicle accidents (n=42; 30.4%) were the main causes of TBI. Most patients had suffered intracranial haemorrhage (n=92; 66.7%), with craniotomies performed in 42.0% (n=58) of all patients to evacuate space-occupying haematomas. Concomitant injuries were infrequent, although 32.6% (n=45) of patients had also suffered chest trauma. The majority of patients had severe brain injury, with 58.7% (n=81) presenting with a documented GCS of ≤8 at the time of ICU admission.

The median lengths of stay in ICU and in hospital were 6 and 16 days, respectively. Outcomes data showed that 65.9% (n=91) of patients survived until the time of discharge from hospital. In terms of neurological outcome at the time of discharge, medical records were incomplete for 67.6% (n=49) of patients, and as such, the GOS of these patients could not be determined. As shown in Table 1, of the remaining 89 patients whose GOS could be determined, 52.8% (n=47/89) were deceased and 7.9% (n=7) were in a persistently vegetative state, while 14.6% (n=13) made a good recovery.

## Discussion

Road accidents and interpersonal injuries were among the top 10 leading causes of death in SA in 2016.^[12]^ Our study similarly found a high mortality in patients who sustained TBI as a result of motor vehicle accidents and assaults. Buitendag *et al*.^[13]^ reported a mortality rate of only 1.83% in patients with penetrating TBI, which contrasts sharply with a mortality rate exceeding 50% in our patients, who primarily sustained blunt trauma to the head rather than penetrating injuries. Less than 15% of the patients had made a good recovery by the time they were discharged from hospital. It therefore could be expected that full-time care and/or supervision would have been required for ~70% of the survivors.

### Study limitations

There were several limitations to our study. It was a retrospective audit of patient hospital files, which were often incomplete in terms of the initial recording of GCS, as well as GCS and functional abilities at the time of discharge from ICU or hospital. A limited number of patients could be included in the study, and the risk of bias should therefore be noted. Since the final cause of death was not determined, mortality could have resulted from concomitant injuries or comorbid conditions, and not necessarily as a result of the TBI. Furthermore, severity of illness scores, such as the APACHE II, were not reviewed, since these were infrequently recorded during the period 2013 - 2017. It would also have been preferable to determine outcomes at 3 months, 6 months and 1 year post-hospitalisation, since improvement in neurological function could have occurred during this time. Since the focus of the current study was in-hospital outcomes, patients or relatives were not contacted to assess long-term neurological outcomes. Lastly, several factors that could have influenced survival, such as pre-hospital care or resuscitation, oxygenation and haemodynamic status, classification of the specific types of TBI, timing and type of neurosurgical interventions, ICU care received and post-ICU hospital care or rehabilitation, were not specifically assessed. Well-designed prospective longitudinal studies should therefore be conducted to assess all the factors that may influence longterm survival or neurological outcome, in order to identify areas of healthcare that can be improved upon.

## Conclusion

Despite the limitations of this study, it highlights the need for prospective studies on the quality of care provided to TBI patients to be conducted in resource-constrained settings. We were able to determine that post hospitalisation care and rehabilitation was required for 7/10 patients surviving TBI. Taking into account the lack of public rehabilitation facilities that is problematic throughout SA,^[9]^ survivors of TBI are not only challenged by the consequences of their injuries, but also deprived of the opportunity to receive appropriate rehabilitation, to improve their quality of life.

The results of this study can inform our future practice in terms of preparing relatives or caregivers of patients with TBI in advance regarding post-hospital discharge care that may be required by patients. Prospective studies with long-term follow-up of patients are now required to determine the extent of neurological recovery post-TBI. More important, however, is to find innovative and pragmatic community-based solutions for the lack of post-hospitalisation rehabilitation facilities in a country where a leading cause of death and disability among young people is TBI.

Our study confirms an exceedingly high mortality and morbidity associated with TBI in young adults. Since the main causes of TBI are road accidents and interpersonal violence, greater awareness and concerted effort from traffic and police authorities are required to reduce the incidence of trauma and injuries. Posthospitalisation discharge rehabilitation and support of patients and their caregivers are lacking, and this requires urgent redress.

## Figures and Tables

**Table:** Table 1. Demographic and clinical information of patients (*N*=138) with TBI treated in ICUs at tertiary hospitals

Variable	Frequency, *n *(%)*	
Age (years), median (range)	30.5 (13 - 70)
Sex	
Male	114 (82.6)
Female	24 (17.4)
Mechanism of injury	
Motor vehicle accident	42 (30.4)
Pedestrian-vehicle accident	20 (14.5)
Assault	57 (41.3)
Fall from height	4 (2.9)
Other	13 (9.4)
Unknown	2 (1.5)
Type of head injury	
Diffuse axonal injury	13 (9.4)
Intracranial bleeding	92 (66.7)
Brain oedema	33 (23.9)
Concomitant injuries	
Chest	45 (32.6)
Abdominal	16 (11.6)
Pelvis	11 (8.0)
Long bone	12 (8.7)
Spinal	23 (16.7)
Facial fractures	21 (15.2)
Other	23 (16.7)
GCS prior to ICU admission	
3 - 8	78 (58.7)
9 - 12	33 (24.8)
13 - 15	22 (16.5)
Unknown	5 (3.6)
Neurosurgical intervention	
None	41 (29.7)
ICP monitor insertion	34 (24.6)
Evacuation of bleed	58 (42.0)
Decompressive craniectomy	29 (21.0)
Other	4 (2.9)
Length of ICU stay (days), median (range)	6 (1 - 146)
Length of hospital stay (days), median (range)	16 (1 - 146)

TBI = traumatic brain injury

ICU = intensive care unit

GCS = Glasgow Coma Scale

ICP = intracranial pressure

^*^Unless otherwise specified.
